# Engagement and Mentorship of Undergraduate Students in Aging Research

**DOI:** 10.1093/geroni/igaa057.1783

**Published:** 2020-12-16

**Authors:** Alyssa Danner, Christina Whitehouse, Melissa O’Connor

**Affiliations:** Villanova University, Villanova, Pennsylvania, United States

## Abstract

Education and mentorship of undergraduate students extends beyond the classroom and clinical setting into research. This presentation will discuss the experience of the student and research mentor as well as provide findings from an important qualitative research study. Through a university funded grant, a student nurse partnered with faculty to investigate the needs and experience of caregivers of recently hospitalized older adults with diabetes. Caregivers play a vital role in caring for older adults, often they do not receive the education necessary to achieve optimal health outcomes. Qualitative interviews of 20 caregivers were conducted. The main themes that emerged from the data were: the role itself, challenges, preparation, and additional comorbid diagnoses. These findings provide an understanding of experience, tasks, and needs of caregivers for older adults with diabetes. This research experience provided exposure and education that is essential to developing future aging research scientists.

